# Association between Four-Level Categorisation of Indoor Exposure and Perceived Indoor Air Quality

**DOI:** 10.3390/ijerph15040679

**Published:** 2018-04-04

**Authors:** Katja Tähtinen, Sanna Lappalainen, Kirsi Karvala, Jouko Remes, Heidi Salonen

**Affiliations:** 1 Department of Civil Engineering, Aalto University, 02150 Espoo, Finland; heidi.salonen@aalto.fi; 2 Finnish Institute of Occupational Health, Healthy Workspaces, P.O. Box 40, 00032 Työterveyslaitos, Finland; sanna.lappalainen@ttl.fi (S.L.); kirsi.karvala@ttl.fi (K.K.); jouko.remes@ttl.fi (J.R.)

**Keywords:** indoor air, impurity sources, categorisation, building investigation, repair urgency, indoor air questionnaire

## Abstract

The aim of this study was to develop and test a tool for assessing urgency of indoor air quality (IAQ) measures. The condition of the 27 buildings were investigated and results were categorized. Statistical test studied the differences between the categories and the employees’ complaints about their work environment. To study the employees’ experiences of the work premises, a validated indoor air (IA) questionnaire was used. This study reveals a multifaceted problem: many factors affecting IAQ may also affect perceived IAQ, making it difficult to separate the impurity sources and ventilation system deficiencies affecting to employee experiences. An examination of the relationship between the categories and perceived IAQ revealed an association between the mould odour perceived by employees and mould detected by the researcher. A weak link was also found between the assessed categories and environmental complaints. However, we cannot make far-reaching conclusions regarding the assessed probability of abnormal IA exposure in the building on the basis of employee experiences. According to the results, categorising tool can partly support the assessment of the urgency for repairs when several factors that affect IAQ are taken into account.

## 1. Introduction

Indoor air quality (IAQ) problems in buildings are common in Finland. In 2015, construction works in Finland totalled about 1.5 million buildings, of which 51% were built before 1980. Their combined value was 17.1 billion €, and of this, renovation construction accounted for 6.8 billion € [1]. The prevalence of significant mould and moisture problems in Finnish building stock has been estimated to be 2.5–26%, depending on the type of building. Cost estimates are 1.2–1.6 billion € [2]. In addition to moisture and mould damage, several other factors and their interactions, such as material emissions [3], ventilation deficiencies [4] and system impurities [5], outdoor and soil impurities [6], human activities in the premises [7] and indoor air (IA) temperature [8] can cause IAQ problems and difficulties for occupants or employees. The ageing of building stock, neglectful maintenance, and building and planning errors are often the cause of IAQ problems and are associated with environmental complaints [9]. Several studies have found that the problems reported in damaged and non-damaged buildings vary according to building types [10,11,12,13]. Different building-related and individual factors, as well as those in the psychosocial environment, affect reported IAQ [14,15,16]. Thus, when examining IAQ problems, it is advisable to assess the problem from a wider perspective, also taking into account the experience of the users of the premises and the psychosocial environment [10,14,15].

Several previous studies have convincingly created methods for identifying, classifying and predicting factors that indicate building IAQ problems [17,18,19,20,21,22,23,24]. In addition, computational, scientific and complex methods and indices have been created from the viewpoint of the building’s moisture and mould damage [17,19,22,25,26,27]. Many of these methods are complicated, and their applicability in the decision-making processes of IAQ is challenging. Several proactive or index-based methods have studied individual sources of IA pollutants and their concentrations, as well as indicators of the properties of the materials and building maintenance that directly or indirectly affect IAQ. The disadvantage of identifying an individual IAQ pollutant is that it does not constitute an overall assessment of the factors affecting IAQ. In these cases, the actual risk and the source of impurities, and their extent, severity and significance to IAQ cannot be concluded and hence, assessment of the impact on the health of the premises’ users is usually impossible. Data from a number of studies are based solely on the problems reported by the residents or users of the buildings, and lack researchers’ investigations and verification of damage in the building and the sources of impurity. A recently published study by Mendell and Kumagai [28] found deficiencies in the methods of observation of building damage.

In order to assess the health significance and the urgency and extent of the measures, property owners, occupational health experts, and safety and healthcare professionals need comprehensive information on the buildings and on the sources and severity of the impurities. IAQ problems may also affect sick leave and work efficiency [29,30], and there is evidence that the repair of moisture and mould damage and the removal of contaminants from buildings reduce respiratory symptoms [12,31] and improve work efficiency and IAQ [32]. Moreover, predictive property management has been estimated to reduce symptoms among premises users [33]. When the extent and severity of IAQ problems are properly assessed, the degree, timing and possible prioritisation of measures can be successfully completed. Properly timed and targeted measures have important implications for the economy, health and well-being.

Previous studies have estimated that researcher surveys of buildings have been more reliable than the damage reports of residents themselves [28,34,35]. It is rarely possible to detect microbial damage in structures through mere observation [28]. Furthermore, measurement of IA contaminants often fails to demonstrate microbial damage in building materials [36,37]. Therefore, indicators of moisture and mould damage should be observed in the buildings [38]. It is important to evaluate the structures from their moisture and thermal aspects, to make the necessary structural openings and to take samples from the structures that are assessed as being at risk and have indications of damage [9,39,40]. A previous study has already determined that the natural ageing of coating materials and excessive moisture on structures causes microbial damage to materials [41], which can also be considered in connection with moisture damage as an indicator of indoor microbial impurities [7,42]. It has been estimated that the classification of pollutants play an important role in assessing exposure in mould-damaged workplaces, and that mould growth in association with moisture damage could be a relevant criterion for the assessment of exposure [43]. In this case, a pragmatic approach is needed for categorising the factors affecting IAQ as a whole and the urgency of the measures from the perspective of building health. 

The aim of this study was to: (i) test and develop the use of a probability of an abnormal IA exposure assessment tool for determining the urgency for the measures from the perspective of building health; (ii) evaluate the relation between the probability of abnormal IA exposure results and workers’ perceived work environment and (iii) assess the relation between ventilation system deficiencies and the perceived work environment. The results did not enable far-reaching conclusions regarding sources of impurities in the building on the basis of employees’ experiences. According to the results, categorising tool can partly support the assessment of the urgency for repairs when several factors that affect IAQ are taken into account.

This paper uses the term probability of abnormal IA exposure, which means a comprehensive categorisation method for deficiencies in building and ventilation systems and other possible IA impurities that affect IAQ.

## 2. Materials and Methods

### 2.1. Materials

The data are from two research and development projects from 2013 to 2014 which investigated the buildings of two Finnish hospital districts. The cooperation agreement of the projects is governed by the obligation of confidentiality. This publication with analyses and writing was made between October 2016 to February 2018. Background information revealed that parts of the buildings had IAQ problems. The study investigated 27 buildings, totaling an area of about 130,000 m^2^, and focused on 111 building floors and hospital sections. The mean building year was 1963. The oldest building was built around the year 1902 and the newest in 2010. All the buildings were of stone in the form of massive brick, concrete or concrete elements with insulation, or different combinations of stone materials.

### 2.2. Building and Ventilation System Investigation

The systematic building investigations covered: (i) construction and architectural plan surveys; (ii) maintenance staff interviews; (iii) examinations and openings of high risk building constructions; (iv) moisture- and mould-damaged range and severity authentications (v) assessments of ventilation systems; (vi) assessments of air leaks from or through damaged constructions; (vii) assessments of air pressure differences; and (viii) assessments of other IA pollutants or pollutant sources in the buildings.

The condition of all the 27 hospital buildings and their floors (111) were investigated carefully and categorised on a scale of 1 to 4 (probability of abnormal IA exposure). Sixteen premises or floors were not in use. We did not categorise these premises, but we did investigate them and take into account the effect of the findings on the IAQ of the surrounding premises. In addition to the age of the ventilation systems, purity, sufficiency and pollution sources were assessed. The same IA researcher group conducted all the building investigations and ventilation system assessments. All the data were analysed by the same multi-professional group of experts, which comprised IA researchers, a construction engineer, an occupational health physician, a microbiologist, and a ventilation specialist.

The building’s technical investigations focused on verify mould and moisture damage and their extent, severity and range in the constructions and on observations of air leaks from or through a damaged construction. In addition, the occurrence of other relevant IA pollution from materials such as man-made vitreous fibres (MMVFs), damaged floor coatings and creosote was assessed, and asbestos was measured. Background information on the buildings included planning, investigation and renovation documents, as well as interviews of the maintenance personnel. The assessments of high-risk building constructions and ventilation systems were based on documents such as construction, ventilation and architectural plans, and required: (i) experimental and theoretical knowledge of heat and moisture transfer in structures and materials; (ii) details of air tight performances in construction joints; (iii) the constructions’ details, materials, sensitivity to moisture damage, and their location in the building; (iv) the typical time of construction and the construction materials used; (v) knowledge of well-known IA pollutants sources such as asbestos and creosote; (vi) details on the theoretical impact of the maintenance history and earlier repairs on the constructions’ functions; and (vii) background information on the ventilation systems and earlier repair and maintenance history.

All ventilation systems were inspected, and the focus was on their age, pollution sources, purity, and ventilation sufficiency. The aim was to detect and identify all significant sources of impurity on the premises. Microbial growth in the structures was primarily verified with microbial analyses, except for cases in which mould growth could be seen in materials or on surfaces. Samples were taken to detect surface moisture in floor and wall structures from nearly all the premises studied. More detailed measurements and sampling were carried out if there was uncertainty regarding the presence of an impurity source or if it was suspected but could not be visually identified. The measurement data will be published in a separate journal article.

### 2.3. Assessment of Probability of Abnormal IA Exposure

The tested method for assessing IA impurity sources and IAQ was based on Finnish authorities’ instructions [44] The National Building Code of Finland [45,46] and earlier studies on the severity of mould and moisture damage [7] and its significance for health [28,47,48,49].

First, the building investigation and ventilation system assessment results were studied and categorised as follows: (i) assessment of the extent and range of mould damage in the constructions (Table 1); (ii) assessment of air leaks from or through damaged constructions and air pressure differences (Table 2); (iii) assessment of ventilation systems and their impact on IA (Table 3); and (iv) assessment of other IA pollutants from the building or from its use (Table 4).

Second, categorised parameters were collected for the final assessment (Table 5) of the probability of abnormal IA exposure. The final assessment was categorised into four levels: (1) probability of abnormal IA exposure unlikely; (2) probability of abnormal IA exposure possible; (3) probability of abnormal IA exposure likely; and (4) probability of abnormal IA exposure very likely.

The final assessment of probability of abnormal IA exposure is presented in Table 1 and Table 2 in accordance with Table 5. The predominant IA impurity source was a determining one. In cases of moisture and mould damage, air leaks through or from damage to IA must be looked at simultaneously with indoor negative pressure. The impact and extent of the problem and the impurity source must always be taken into account in the assessment. For example, the whole building is not in category very likely if the impurity sources are only in the basement and they are not influencing to the upper floors. The ventilation systems’ impact on IA (Table 3) and the IA measurement results were evaluated at the same time with the final assessment of the probability of abnormal IA exposure (Table 5).

### 2.4. Measurements and Sampling in Buildings

In building and IAQ measurements and interpretation of measurement results we followed the Finnish national instructions to the monitoring of health-related conditions of housing and other residential buildings [44]. Microbial growth in the structures was primarily verified with microbial analyses, except for cases in which mould growth could be seen in materials or on surfaces. Materials microbial growth and contamination were verified with microbial analyses in according to ISO 16000-19 [51]. Structural humidity and incision moisture measurements between floor surfaces and concreate were measured with Vaisala HMI41, HMP42, HMP40 devices (Vaisala Oy, Vantaa, Finland). Surfaces moisture detection were measured with Moisture Encounter Plus (Tramex Ltd., County Wicklow, Ireland) and Gann Hydrometer RTU600, B50 (Gann Mess-u., Regeltechnik GmbH, Gerlingen, Germany). Measurements were conducted concreate or other materials humidity in accordance with the Finnish guidance for concrete floor structures, moisture management and coating [52]. Sampling of asbestos were made according to ISO 16000-7 standard [53]. Volatile organic compound (VOC) sampling were carried out according to ISO 16000-6 standard [54]. The air flow tester (Dräger smoke, Drägerwerk AG & Co., Lübeck, Germany) were used to visualize air flows, rates and pressure. Ventilation rates and the carbon dioxide content were measured in according to ISO 16000-26 standard [55]. Room temperature and indoor air humidity and air pressure differences were measured (Swema 3000d, Swema AB, Stockholm, Sweden) if needed and measured in according to SFS-EN 12599 standard [56].

### 2.5. Employees’ Experience of Indoor Air Quality

To study the employees’ experiences of the work premises, validated and frequently used IA questionnaire was used, which is based on Örebro’s [57] indoor climate questionnaire [11,58]. The questionnaire was sent to 3608 hospital employees, of whom 2669 responded. The total response rate was 74% with a range of 51 to 93%. The IA questionnaire groups comprised employees working in the same workspaces in the same building and under technically similar circumstances. Forty IA questionnaire groups were selected for this study, totalling 1587 respondents. One selection criterion was that the IAQ groups should be in such premises in which a probability of abnormal IA exposure assessment had been performed. Cases in which the questionnaire had been carried out among employees in the whole building as one group, and in which the probability of abnormal IA exposure assessment concerned different sections of the building, were excluded. This is a questionnaire based study, in which participation was voluntary and since there was no intervention on individuals, the Finnish legislation does not require ethics committee handling.

### 2.6. Statistical Analyses

Statistical analyses were carried out using IBM SPSS Statistics program 24.0 (SPSS Finland Oy, Espoo, Finland) with a statistically significant level of *p* < 0.05. The statistical analysis used weighted averages of group response rates. The Mann-Whitney U test studied the differences between the probability of abnormal IA exposure categories (unlikely, possible, likely and very likely) and the employees’ complaints about their work environment. This test also compared the difference between two groups ‘yes’ and ‘no’ (categorised according to building investigations findings) ventilation adequacy, ventilation MMVF sources, ventilation moisture problems, and ventilation lifespan having expired and employees’ complaints about their work environment.

## 3. Results

### 3.1. Probability of Abnormal Indoor Air Exposure

The probability of abnormal IA exposure was likely on 39% of the floors of the building and very likely on 13%, meaning that these floors had wide moisture and mould damage in their structures together with air leaks from damaged materials to the IA (Table 6).

**Figure 1:**
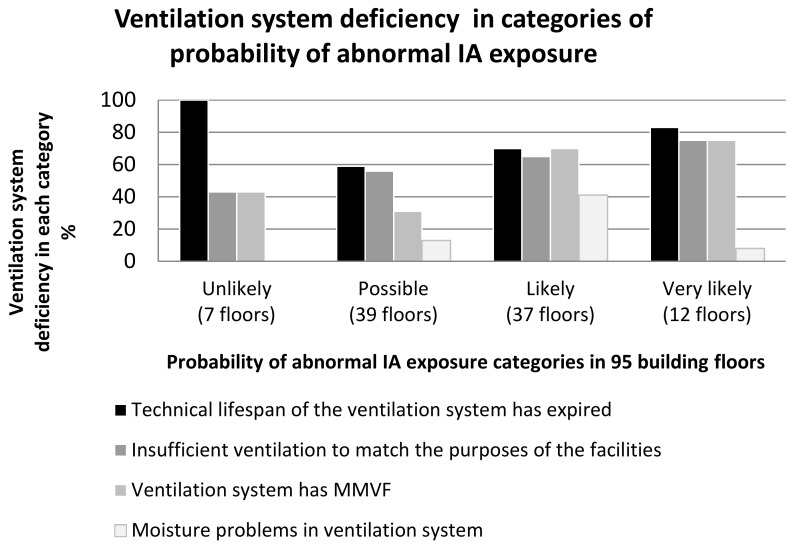
Categories of probability of abnormal indoor air (IA) exposure and ventilation system deficiency.

The probability of abnormal IA exposure was mostly assessed as very likely in basements in which the building structures faced the ground. The most typical moisture and mould damage in the building were in structures that faced the ground. Technology channels (a horizontal or vertical case or structure in which heating or sewer pipes) in the premises were detected in 11 building sections of the selected 40. The structure materials of these channels were invariably moisture and mould damaged and air leaked from the channels to the IA. In addition, in buildings that were over 50 years old, the intermediate floors with double concrete structures and organic insulation inside the structure were frequently found to be damaged. The higher (more abnormal) the probability of abnormal IA exposure category was assessed as being, the more insufficient the ventilation was, or the ventilation did not match the purposes of the facilities (Figure 1). The survey materials contained several different ventilation systems and machines that served different parts of the building. The maintenance, repair, reliability and age of the ventilation systems varied considerably across the floors of even one building. The technical lifespan of the ventilation system had mostly expired in the category assessed as unlikely. When the lifespan of the ventilation system had expired, an MMVF source was also found in the ventilation system.

### 3.2. Relation of Probability of Abnormal Indoor Air Exposure and Employees’ Complaints

**Figure 2:**
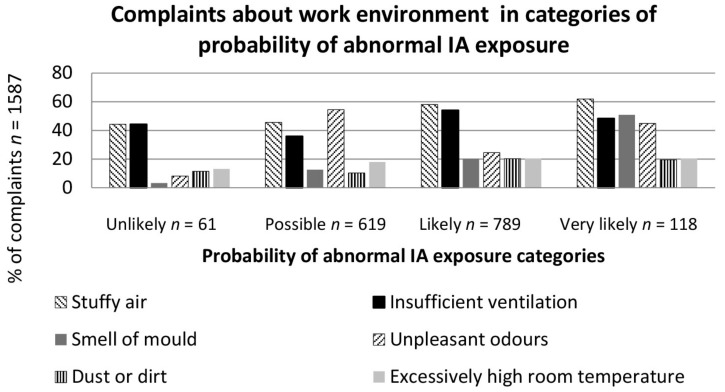
Categories of probability of abnormal IA exposure and employees’ complaints about their work environment.

In the floors assessed for probability of abnormal IA exposure, discomfort from insufficient ventilation was perceived weekly by 30% of the respondents, stuffy air by 26%, unpleasant odours by 14%, excessively high room temperature by 11%, smell of mould by 11%, and dirt and dust by 9%. The more probable the abnormal IA exposure was assessed as being, the more the employees’ perceived stuffy air (Figure 2).

The more mould damage that was detected, the more employees observed the smell of mould (Figure 2). The smell of mould was the second most common cause of discomfort in the very likely category (Figure 2), meaning that considerable moisture and mould damage was found in several building structures. Dirt and dust were complained about the most in the likely category (Figure 2). Excessively high room temperature was complained about evenly in each category (Figure 2). Unpleasant odours were complained about mostly in the possible category (Figure 2). The higher the probability of abnormal IA exposure was assessed as being, the more often the smell of mould and stuffy air was reported (Figure 2).

In the floors assessed for probability of abnormal IA exposure, 51% of the employees reported insufficient ventilation in the premises for which the IAQ researcher assessed ventilation as insufficient and as not matching the purpose of the premises. In these premises, the probability of abnormal IA exposure was most commonly assessed as likely. However, in the premises in which the IAQ researcher assessed the ventilation as sufficiently good, 42% of employees perceived the ventilation as insufficient. In the latter premises, the probability of abnormal IA exposure was most commonly assessed as likely, because of moisture and mould damage.

On the floors assessed for probability of abnormal IA exposure, 27% of the employees reported the smell of mould and 29% of the employees complained of other unpleasant odours. The complaints of mould odour were reported in the premises in which the IAQ researcher observed the smell of mould, and the probability of abnormal IA exposure was most commonly assessed as likely. Complaints of unpleasant odours were reported in the premises for which the probability of abnormal IA exposure was most commonly assessed as possible.

An analysis of the differences between the probability of abnormal IA exposure groups (unlikely, possible, likely, very likely) and the employees’ weekly complaints revealed significant difference (*p* = 0.042) between the “unlikely” and “likely” groups regarding smell of mould weekly. No statistically significant differences were found between the other groups’ means (abnormal IA exposure unlikely, possible, likely, very likely) regarding employees’ weekly complaints of work environment.

The differences between the two groups ‘yes’ and ‘no’ (categorised according to building investigations findings) for ventilation system lifespan has expired, insufficient ventilation or ventilation system does not match the purposes of the facilities, moisture problem in ventilation system and MMVF source in ventilation system and employees’ weekly complaints of environmental problems were studied. The following statistically significant differences between the two groups’ means were found (Table 7): MMVF source in ventilation system and stuffy air, insufficient ventilation and dirt or dust.

Statistically significant differences were also found between the following studied groups’ means: expired technical lifespan of the ventilation system and weekly reported stuffy air, insufficient ventilation, smell of mould, dirt or dust, and excessively high room temperature. In contrast, no statistically significant associations were found between employees’ complaints about the work environment and moisture problems in the ventilation system. No statistically significant associations were found between employees’ complaints regarding the work environment and insufficient ventilation or the ventilation system not matching the purposes of the facilities (Table 7).

## 4. Discussion

This was the first study in Finland to use a comprehensive, systematic method of categorising impurity sources from buildings and their impact on IAQ. We know of no previous studies that have as extensively taken both building technology and ventilation-related factors that affect IAQ into account.

The strength of this research was its systematic and comprehensive building surveys, which were carried out by researchers, and IAQ questionnaires for the users of the premises. The IAQ questionnaire group division followed the same distribution as the assessment of probability of abnormal IA exposure. The employees did not know the results of the assessment of probability of abnormal IA exposure prior to responding to the IAQ surveys, which contributes to the reliability of the study.

The results support previous observations of the connection between the age and moisture of a building and mould [59]. The building stock of the research was mostly over 50 years old. The technical lifespan of the building materials used had already been exceeded, which had led to deterioration of the technical properties of the structures and allowed moisture access to structures. 

Knowledge of building physics and materials technology and construction techniques have changed over the decades in Finland. Therefore, the plans, building materials and building implementations of buildings aged over 50 years, which were the object of this study, are also often risky [9]. In the surveyed buildings, more than 50% of the examined building floors had extensive microbial or moisture damage in one or two building blocks. Earlier repairs to the buildings had not generally taken into account the sources of the contaminants inside the structures or the connections between these sources and the IA and the airtightness of the structures, which had resulted in the presence of contaminants in even refurbished buildings.

According to the results, workers perceived mould odour in the same parts of the building as those in which the IAQ researcher made the mould odour observations and premises or where the floor of the building had extensive moisture and or mould damage. These results support earlier studies in that the mould odour and moisture damage were connected [17,26,28,60]. Several studies have found that asthma and respiratory symptoms are linked to moisture and mould damage in buildings [28,47,48,59]. Among qualitative indicators of dampness and mould, mould odour has the strongest associations with health effects [61]. Thus, mould odour observations by the users of premises should always be taken seriously and should lead to more detailed studies to determine the origin of the odour.

In previous studies, inadequate ventilation has associated with poor IAQ [62,63,64,65,66,67]. This supports the results of this research. Employees experienced the most dissatisfaction with weekly inadequate ventilation and the second most dissatisfaction with stuffy air. The worse the probability of abnormal IA exposure was estimated to be, the more problems and impurity sources appeared in the ventilation system. On the basis of the results, the poor condition of a building also indicates deficiency in ventilation. Employees also experienced inadequate ventilation even in the premises in which the IA researcher had assessed the ventilation as adequate for the use of the premises. However, it must be taken into account that there was extensive mould damage to the structures in these areas which may also have impact to the experience of poor IAQ. Previous research has found similar results [13], estimating that building mould damage increases the perception of stuffy air. MMVF in the ventilation system may cause the presence of particulate fibres both in IA and on surfaces, and may cause upper respiratory irritation and skin symptoms among the users of the premises [3,63,68,69]. This study found a relation between the presence of MMVF sources in the ventilation system and the perceived IAQ. It also found an association between the age of the ventilation system and the perceived IAQ. Therefore, on the basis of the work environment observations of the users of the premises, it is not possible to distinguish problems in IAQ when the ventilation system is an MMVF source and the system has exceeded its technical lifespan. As ventilation deficiencies were also observed in premises in which the probability of abnormal IA exposure was assessed as being likely or very likely, the experience of the users of the premises could be due to inadequate ventilation and stuffy air from a lack of ventilation and or other sources of IA pollutants in the premises.

As a whole, it seems that the more IA impurity sources that were found in the building, the more the users of the premises experienced poor IAQ. There were no statistical differences between the probability of abnormal IA exposure categories and perceived IAQ, but when the probability of abnormal IA exposure categories was considered higher (more abnormal), there were more work-related complaints. The results of this research on the work environment survey may not be unambiguous, because many factors can be influenced by a person’s experience [10,16,70,71] and experiences may differ from one person to another. The results of this research suggest an agreement between the mouldy odour found by inspectors and that experienced by employees working in the premises. Other IAQ questions on the questionnaire may leave more room for interpretation.

The probability of abnormal IA exposure method was used to systematically and objectively rank the results of the building investigations and the other factors influencing the IA in an overall assessment. The method only describes factors affecting the IA conditions of the building; it does not describe the implications of risk assessment, such as the severity of harm or the symptoms of the premises users. In addition, the method is very pragmatic and is always based on strong technical expertise in building technology and IA. The criteria for the probability of abnormal IA exposure are guidelines only, which means that the assessment also involves a researcher’s subjective view. All IA pollutants cannot be measured and for many IA pollutants, no health-based limit values have been set for non-industrial buildings [28,48]. However, although the method depends on the researcher’s competence and interpretation, and the model is partly based on national guidelines, it is nevertheless a pragmatic tool and a good basis for further developing the categorised exposure scenario.

Workplaces may also have other factors that affect the perceived indoor environment, and individual factors may also play a role. Thus, perceptions of the work environment are not directly proportional to the identified technical findings in buildings and in IAQ [16]. The results did not suggest significant enough associations between the probability of abnormal IA exposure categories and perceived IAQ, hence this research does not assert the validation of the method.

## 5. Conclusions

This study tested and developed a comprehensive, systematic and detailed method for categorising impurity sources in buildings and their impact on IAQ. The tested method was used to evaluate a demanding and complex entity of building conditions from the perspective of building health. In addition, the differences between categorised building conditions and the results of employees’ complaints about their work environment was examined. This study reveals a multifaceted problem: many factors affecting IAQ may also affect perceived IAQ, making it difficult to separate the impurity sources and ventilation system deficiencies affecting to employee experiences. The results did not enable far-reaching conclusions regarding assessed probability of abnormal IA exposure on the basis of employees’ experiences. For comprehensive assessment of IA circumstances, valid researchers should always investigate building technology, the condition of the ventilation system, impurity sources, and the cause of mould odour at workplaces with suspected IAQ problems. This method enables the systematic detection of many factors that affect IAQ and hence generates categorised information that might be used to support health assessments of the users of the premises. The method is promising, but further studies need to assess the relationship between the probability of abnormal IA exposure categories and employees’ health, symptoms and stress factors.

## Figures and Tables

**Table 1:** Main criteria for assessing the extent and range of moisture and mould damage in constructions.

The Extent and Range of Moisture and Mould Damage in Constructions	
1. No mould damage in structures	Building or premises have no mould-damaged structures or previous local moisture damage in structures has been repaired.
2. Slight, limited mould damage in structures	Building or premises have some mould-damaged constructions. Mould-damaged structure type is not widespread in building and repairs are easily definable (less than 1 m^2^).
3. Extensive mould damage in structures	Wide-ranging mould-damaged structure in building or premises. There are recurrent damages in the type of the structure. Repairs are significant and affect a large part of the (one) structure in the building or premises (e.g., whole base floor structure) and repair planning requires structural engineering in building physics.
4. Extensive mould damage in several structures. Extent of repair is significant.	Building or premises has a great deal of extensive mould damage in several structures. There are recurrent damages in the type of the structures. Extent of repairs is significant and affects several structures in the building or premises (e.g., whole façade and whole base floor) and repair planning requires structural engineering in building physics.

**Table 2:** Main criteria for assessing air leaks and air pressure in buildings or premises.

Air Leaks and Air Pressure in Buildings or Premises	
1. No air leaks from or through damaged structures, and air pressure differences	The ventilation system can be controlled by indoor air pressure difference from the building envelope [46], (IA pressure difference commonly −2–2 Pa). The air-tightness of the building or premises is good (n_50_ < 1–2) ^1^ [50].
2. A few or single air leaks from or through damaged structures or from surrounding premises	A few or single air leaks from structures, caused by single cable entries through structures or single non-tight junctions of the structures. Ventilation system can be controlled by indoor air pressure difference from the building envelope [46], (IA pressure difference commonly −2–2 Pa). Air pressure differences do not change significantly between day and night. The air-tightness of the building or premises is slightly risky (n_50_ = 2–3) ^1^ [50].
3. Air leaks from or through damaged structures are regular and recurrent	Air leaks from or through damaged structures or from surrounding premises’ structures that have moisture or mould damaged materials are regular and recurrent. Air pressure differences change, and occasionally there is negative pressure (commonly 2–15 Pa) in the premises or building and/or the air-tightness of the building or premises is risky (n_50_ = 3–4) ^1^ [50].
4. Air leaks from or through damaged structures are regular and recurrent, negative pressure is significant in the premises and or air-tightness is risky	Air leaks from or through damaged structures are regular in structures. Air pressure differences change significantly and there is negative pressure (commonly over 15 Pa) in the premises or building during both day and night and/or the air-tightness of the building or premises is very risky (n_50_ > 4) ^1^ [50].

^1^ n_50_ (1/h) = air leakage rate at 50 Pa. IA: indoor air.

**Table 3:** Main criteria for assessing impact of ventilation systems on indoor air quality (IAQ). The category “Well-balanced and effective ventilation contributes to good indoor air quality” is achieved only if all options are selected.

Well-Balanced and Effective Ventilation Contributes to Good Indoor Air Quality	Poor, Inoperative or Incorrectly Rated Ventilation System Can Reduce Indoor Air Quality
Air flows in premises correspond to Finnish guideline values and regulations (6 dm^3^/s per person) [44]. Ventilation system has no sources of indoor air impurity. Filtering level (F7/G4) of ventilation system’s supply air corresponds to Finnish guidelines and regulations [44]. Condition of ventilation system is good, and the system is maintained regularly.	Air flows in premises do not correspond to Finnish regulations or guideline values. Ventilation system has man-made vitreous fibres (MMVF) impurity sources in machinery and/or on duct materials. Ventilation system materials contain asbestos and asbestos has been found on duct surfaces or on surfaces of the premises (the occurrence of asbestos fibres in dust accumulated on surfaces indicates that the action limit has been exceeded [44]), or fibres have been found in indoor air (the indoor concentration of asbestos fibres may not exceed 0.01 fibres/cm^3^ [44]). Ventilation system has water- or mould damaged materials or impurity sources. Ventilation system’s condition is poor (increased need for maintenance), or its functioning is uncertain. Ventilation system maintenance is not regular, and the system’s parts are dirty and dusty.

**Table 4:** National maximum limit values for IA concentrations, microbial growth on building material and MMVF and asbestos in dust. Evaluation of abnormal indoor microbial sources also includes the identification of micro-organisms (genus/species/groups). With regard to the air sample (microbial), there shall also be other evidence of exceeding the limit value in addition of IA microbial concentration. The limit value for the total indoor concentration of a single organic compound determined as toluene equivalent is 50 µg/m^3^, exceptions are presented in this table [44]. Measurements and sampling methods are shown in the Section 2.4.

Indoor Air Pollutant	Limit Value
Asbestos in indoor air	0.01 fibres/cm^3^
Asbestos in dust	0 fibres/cm^3^
Man-made vitreous fibres in dust (accumulated on surfaces in two weeks)	0.2 fibres/cm^2^
Carbon monoxide	7 mg/m^3^
Fungal spores, indoor air sample (a 6-stage impactor)	50–500 ^b^ cfu/m^3^
Fungal spores, material sample	10,000 cfu/g
Total indoor concentration of volatile organic compounds (TVOC) ^a^	400 µg/m^3^
2,2,4-Trimethyl-1,3-pentanediol diisobutyrate (TXIB) ^a^	10 µg/m^3^
2-Ethyl-1-hexanol (2E1H) ^a^	10 µg/m^3^
Naphtalene ^a^	10 µg/m^3^, odour may not occur
Styrene ^a^	40 µg/m^3^

^a^ Determined as toluene equivalent; ^b^ The limit value varies depending on the type of premises (e.g., hospital and office 50 cfu/m^3^ (reference value); school and residence 500 cfu/m^3^ (limit value)). Cfu: colony forming unit.

**Table 5:** Main criteria and categories for assessing probability of abnormal IA exposure in buildings. In cases of moisture and mould damage, air leaks through or from damage to IA must be looked at simultaneously with indoor negative pressure. The predominant IA impurity source is a determining one. National maximum limit values for IA concentrations, microbial growth on building material and MMVF and asbestos in dust are shown in the Table 4.

The Categories	The Main Criteria for Assessing Probability of Abnormal IA Exposure in Buildings.
Unlikely	No moisture or mould damage in structures. No air leaks from or through damaged structures. Ventilation system can be controlled by indoor pressure difference from the building envelope. Room acoustic materials and ventilation system have no man-made vitreous fibres (MMVF) sources. Indoor air quality corresponds to national reference values and guidelines set for the premises.
Possible	Mould-damaged structure type is not widespread in building and repairs are easily definable (less than 1 m^2^). A few or single air leaks from or through damaged structures or from surrounding premises. Room acoustic materials or ventilation system have MMVF sources and fibres may end up in the indoor air or on surfaces ^1^. Concrete floor has extensive moisture, which can cause water vapour damage to permeable floor coating (emissions) ^1^. Indoor air quality does not correspond to national reference values or the guidelines set for the premises, and indoor air impurity source has been identified ^1^.
Likely	Building or premises have widespread mould-damaged structure. Repairs are significant and affect a large part of the (one) structure, in the building or premises, e.g., whole base floor structure. There are recurrent damages in the type of the structure. Air leaks from or through damaged structure or from surrounding premises and moisture or mould damaged materials are regular and recurrent in structure, occasionally there is negative pressure in the premises and/or air-tightness is risky. Indoor air quality does not correspond to national reference values or the guidelines set for the premises, and indoor air impurity source has been identified ^1^. Creosote has been used in the structure and air leaks into the indoor air from the structure. There is also a notable smell of creosote (e.g., naphtalene) in the indoor air ^1^.
Very likely	The building or premises has a great deal of extensive mould damage in several structures. The extent of repairs is significant and affects several structures in the building or premises e.g., whole façade and whole base floor. There are recurrent damages in the type of the structures. Air leaks from or through damaged structures are regular and recurrent, negative pressure is significant in the premises and/or air-tightness is very risky. Indoor air quality does not correspond to national reference values or the guidelines set for the premises, and indoor air impurity source has been identified ^1^. Creosote has been used in the structures and air leaks into the indoor air from the structures. In addition, concentrations of polycyclic aromatic hydrocarbons (PAH) or separate components exceed the set national values and guidelines^1^. Dust sample tests have found asbestos fibres in the premises, and the pollution source has been defined ^1^. Indoor radon concentrations exceed the set national values and guidelines (400 Bq/m^3^ [46]) ^1^.

^1^ The extent and impact of the problem and impurity source must be taken into account in the assessment.

**Table 6:** The probability of abnormal IA exposure was assessed in 95 building floors. Probability of abnormal AI exposure categories is based on the method described in Section 2.2 and Section 2.3.

Assessed Probability of Abnormal IA Exposure in Buildings				
Probability of Abnormal IA Exposure	Unlikely	Possible	Likely	Very Likely
Number of floors (*n* = 95)	7	39	37	12
Building floors %	7	41	39	13

**Table 7:** Statistical differences (*p*-value) between employees’ weekly complaints about their work environment and ventilation factors (yes/no) studied.

Weekly Complaints about Work Environment	Technical Lifespan of Ventilation System Has Expired	Moisture Problem in Ventilation System	MMVF Source in Ventilation System	Ventilation System Does Not Match Purposes of Facilities
Stuffy air	0.011	NS	0.006	NS
Insufficient ventilation	0.008	NS	0.002	NS
Smell of mould	0.004	NS	-	NS
Unpleasant odour	NS	NS	-	NS
Dirt or dust	0.022	NS	0.003	NS
Excessively high room temperature	0.043	NS	-	NS

NS: no significant.

## Abbreviations

The following abbreviations are uses in this manuscript:

IAQ	Indoor air quality
IA	Indoor air
FIOH	Finnish Institute of Occupational Health
MMVF	Man-made vitreous fibres
PAH	Polycyclic aromatic hydrocarbon
TBIX	2,2,4-Trimethyl-1,3-pentanediol di-isobutyrate
TVOC	Total indoor concentration of volatile organic compounds
VOC	Volatile organic compound
2E1H	2-Ethyl-1-hexanol
